# Neuronal AMP-activated protein kinase hyper-activation induces synaptic loss by an autophagy-mediated process

**DOI:** 10.1038/s41419-019-1464-x

**Published:** 2019-03-04

**Authors:** Manon Domise, Florent Sauvé, Sébastien Didier, Raphaëlle Caillerez, Séverine Bégard, Sébastien Carrier, Morvane Colin, Claudia Marinangeli, Luc Buée, Valérie Vingtdeux

**Affiliations:** Université de Lille, Inserm, CHU-Lille, UMR-S1172 – JPArc – Centre de Recherche Jean-Pierre AUBERT, F-59000 Lille, France

## Abstract

Alzheimer’s disease (AD) is a neurodegenerative disorder characterized by synaptic loss that leads to the development of cognitive deficits. Synapses are neuronal structures that play a crucial role in memory formation and are known to consume most of the energy used in the brain. Interestingly, AMP-activated protein kinase (AMPK), the main intracellular energy sensor, is hyper-activated in degenerating neurons in several neurodegenerative diseases, including AD. In this context, we asked whether AMPK hyper-activation could influence synapses' integrity and function. AMPK hyper-activation in differentiated primary neurons led to a time-dependent decrease in pre- and post-synaptic markers, which was accompanied by a reduction in synapses number and a loss of neuronal networks functionality. The loss of post-synaptic proteins was mediated by an AMPK-regulated autophagy-dependent pathway. Finally, this process was also observed in vivo, where AMPK hyper-activation primed synaptic loss. Overall, our data demonstrate that during energetic stress condition, AMPK might play a fundamental role in the maintenance of synaptic integrity, at least in part through the regulation of autophagy. Thus, AMPK might represent a potential link between energetic failure and synaptic integrity in neurodegenerative conditions such as AD.

## Background

Alzheimer’s disease (AD) is the leading cause of dementia in the elderly. AD is characterized at the histopathological level by the presence of two lesions known as senile plaques and neurofibrillary tangles that are caused by the aggregation of β-amyloid peptides and tau proteins, respectively. AD symptoms include progressive memory and cognitive functions impairment, which result from synaptic loss that occurs during the disease progression^1,2^. Synaptic loss is indeed largely reported both in post-mortem human AD brain tissues^3^, as well as in brain of AD mouse models^4^. Interestingly, a recent study investigated synaptic density in vivo in AD patients with synaptic vesicle glycoprotein 2A (SV2A, a vesicle-associated membrane protein expressed in all synapses) positron emission tomographic (PET) imaging. This study showed that AD patients displayed a reduction in the SV2A-PET signal in the hippocampus, a decrease that remained significant even after correction for atrophy and that correlated with episodic memory score^5^.

Interestingly, synapses represent the most energy-requiring structures in the brain^6,7^. Energy is provided as ATP, synthesis of which, either driven by glycolysis or mitochondrial respiration, is required to support synaptic functions^8–10^. It is well known that brain energy deficits lead to cognitive impairments and contribute to neurodegeneration. For instance, brain hypometabolism in AD has been reported in many studies and was recently stated to be one of the best predictive biomarkers of this neuropathology^11^. Hence, energy control and homeostasis regulation is crucial for neuronal functionality.

AMP-activated protein kinase (AMPK) is the main sensor and regulator of cell metabolism. AMPK is an heterotrimer composed of one catalytic subunit α (α1, α2), one scaffolding subunit β (β1, β2), and one regulatory subunit γ (γ1, γ2, and γ3), which are all encoded by distinct genes. This kinase is expressed in many peripheral tissues and in the brain where it is mainly expressed in neuronal cells^12^. Once activated by phosphorylation on the α subunit residue Thr172, AMPK inhibits anabolic pathways and stimulates catabolic pathways such as autophagy (for review^13^). Indeed, AMPK directly phosphorylates the autophagy initiator ULK1 (Unc-51-like kinase 1)^14,15^, and Raptor which inhibits the mTORC1 complex consequently switching on autophagy^16^. Importantly, AMPK was reported to be hyper-activated in the neurons of AD patient’s^17^ as well as in Parkinson’s and Huntington’s^18^. In AD, AMPK signaling pathway was proposed to be involved in the degradation of β-amyloid peptides^19,20^ and in tau phosphorylation^17,21,22^. Moreover, AMPK can be activated by β-amyloid peptides to mediate their synaptotoxic effects through tau phosphorylation^23^. Altogether, these studies strongly suggest that AMPK could be an upstream driver in AD progression.

Here, we investigated whether AMPK hyper-activation, as observed in AD, could have any impact on synapses' integrity and function. We showed using primary neuronal cultures that AMPK hyper-activation, induced either pharmacologically or genetically, led to a loss of synapses that impaired neuronal networks functionality. In addition, we provided evidences that post-synaptic proteins were eliminated through AMPK-mediated activation of the autophagic flux. Finally, we showed that this process was also observed in vivo, where AMPK hyper-activation also primed synaptic loss.

## Methods

### Antibodies and reagents

Antibodies directed against AMPKα1/α2 (#2603), ACC (#3676), phospho-ACC-Ser79 (#3661), PSD-95 (#2507), GluA1 (#13185), Raptor (#2280), phospho-Raptor-Ser792 (#2083), ULK1 (#8054), phospho-ULK1-Ser555 (#5869), SQSTM1/p62 (#5114), and LC3B (#2775) were obtained from Cell Signaling Technology. Anti-AMPKα2 (AF2850) was from R&D Systems. Anti-phospho-AMPK-Thr172 (sc-33524), synapsin Ia/IIb (sc-376622), Homer 1bc (sc-25271), synaptophysin (SYP) (sc-17750), GluN1 (NMDA zeta1; sc-1467), and SNAP25 (sc-376713) were from Santa Cruz Biotechnology. Anti-actin (#612656) and Munc (#610336) antibodies were from BD Transduction Laboratory. MAP2 (#M1406) antibody was from Sigma. SMI-312 (#837904) antibody was from Biolegend. AICAR and MRT68921 were purchased from Tocris, GSK621 was from Selleckchem, and Bafilomycin A1 was from Cell Signaling.

### Animals

All animal experiments were performed according to procedures approved by the local Animal Ethical Committee following European standards for the care and use of laboratory animal (agreement APAFIS#4689-2016032315498524 v5 from CEEA75, Lille, France).

### Surgical procedures and injections

Hippocampal surgeries were performed as described^24,25^. Briefly, intracerebral injections of PBS/BSA or PBS/BSA 1%/CA-AMPK (constitutively active form of AMPK) were performed into the hippocampus of anesthetized (100 mg/kg of ketamine and 10 mg/kg of xylazine, i.p.) 10-weeks-old male C57BL/6J mice at the following stereotaxic coordinates relative to the bregma: −2.2 mm anteroposterior [AP], ±1.4 mm mediolateral [ML], and −2.1 mm dorsoventral [DV] according to the Paxinos and Franklin mouse brain atlas. These injections consisted to deliver 400 ng of viral particles diluted in PBS/BSA 1% using a 10-μl glass syringe with a fixed needle (Hamilton; Dutscher, Brumath, France) at a rate of 0.25 μl/min.

### Primary neuronal culture and treatments

Primary neurons were obtained from C57BL/6 wild-type mice as described previously^22^. Fetuses were obtained from females that were sacrificed at 18.5 days of gestation. Forebrains were dissected in ice-cold Hank’s balanced salt solution containing 0.5% w/v d-glucose and 25 mM Hepes under a dissection microscope. Dissociation was carried out mechanically in ice-cold dissection medium containing 1.3 U/ml papain, 0.96 U/ml dispase II, and 500 U/ml DNase and by incubation at 37 °C twice for 10 min. The cells were then spun down at 220×*g* for 5 min at 4 °C, resuspended in Neurobasal medium supplemented with 2% B27, 1 mM sodium pyruvate, 100 U/ml penicillin, 100 µg/ml streptomycin, and 2 mM Glutamax (Invitrogen), filtered through a 40-µm cell strainer, counted, and plated on poly-l-ornithine- and laminin-coated plates at a density of about 10^6^ cells/well. The culture medium was fed by adding new medium (1:3 of starting volume) every 3 days until the end of the culture period. Drug treatments were realized directly in the conditioned media.

### Constructs and production of lentiviral vectors

AMPKα2(1-312)-pLenti construct encoded the constitutive active (CA) truncated form of AMPK ending at position 312 as previously described^10^. FUGW-PK-hLC3 (mKate2-LC3) and FUGW-PK-hLC3ΔG were a gift from Dr. Isei Tanida (Addgene plasmids #61460 and #61461)^26^. The production of lentiviral vectors (LV) batches was as previously described^25^. Primary neurons were infected with LV at 17 days in vitro (DIV) and were used for experiments 7 days after LV infections.

### Cell-stress array

For the cell-stress assay, array membranes were incubated with cell lysates (250 µg of total proteins per array) and subsequently processed according to the manufacturer’s instructions (R&D systems).

### Cytotoxicity assay

Cell toxicity was assessed by the measurement of lactate dehydrogenase (LDH) release as per manufacturer’s instructions (CytoTox 96^®^ Non-Radioactive Cytotoxicity Assay, Promega). Absorbance measurements were obtained using a SpectraMax^®^ i3 (Molecular Devices, Sunnyvale, CA 94089, USA).

### Immunocytofluorescence

Neurons grown on poly-d-lysine and laminin-coated glass coverslips were fixed in 4% paraformaldehyde and permeabilized using 0.25% Triton X-100 before blocking in 1% BSA for 1 h at RT. Neurons were then incubated with primary antibodies directed against MAP2 (1:200 dilution), SMI-312 (1:500 dilution), SYP (1:1000 dilution), and the post-synaptic density (PSD) protein-95 (PSD-95, 1:500 dilution) O/N at 4 °C, followed with anti-IgG secondary antibodies coupled to Alexa Fluor 350, 488, or 568 (Invitrogen). Nuclei were visualized with DAPI. Images were acquired on a Zeiss Axio Imager Z2 microscope (Carl Zeiss, Germany) equipped with a Hamamatsu ORCA-Flash4.0 digital camera (Hamamatsu Photonics, Japan) and ApoTome.2 system (Carl Zeiss, Germany).

### Puncta analyses

After performing immunofluorescence with antibodies directed against the pre-synaptic marker SYP and the post-synaptic marker PSD-95, images were acquired on a Zeiss Axio Imager Z2 microscope (Carl Zeiss, Germany) using an oil immersion 63× objective. Neurons that were at least two cell diameters away from others neurons were selected in order to avoid bias in the synapses quantification. Synapses number was then quantified by the colocalization of puncta between SYP and PSD-95. The number of these puncta were quantified using the plugin Puncta Analyzer from Image J (NIH) (written by Bary Wark) as described by Ippolito and Eroglu^27^. Briefly, regions of interest (ROI) were selected around the soma of the neuron of interest. The background values were subtracted and the threshold was adjusted manually for each channel and was the same within experiments. The minimum puncta size and the rolling ball radius were set to 4 pixels and 50, respectively. At least three independent experiments were performed.

### Western blot

Cell extracts were separated by SDS-PAGE and transferred to nitrocellulose membranes as described previously^22^. The membranes were probed with the antibodies listed above and analyzed by enhanced chemiluminescence detection.

### Multi-electrode arrays

Mouse primary neurons were plated on poly-d-lysine and laminin-coated multi-electrode arrays (MEA) at a density of 40,000 neurons by MEA and cultured at 37 °C with 5% CO_2_. Neuronal electrical activity was extracellularly recorded for 5 min after 15 min of acclimatization, using an MEA60 setup (Multi Channel Systems, Germany). Recorded signals are amplified by a filter amplifier and sent to the data acquisition card for conversion of analog signals to digital data streams. The culture medium temperature was maintained at 37 °C during the recordings, thanks to a thermal element. MEA are composed of 60 electrodes (30 μm diameter, 200 μm spaced) made of titanium nitride, which are aligned in an 8 × 8 grid. Multi Channels Experimenter software was used to display the recorded signals (spikes) in real time and save them. All data were analyzed with Neuroexplorer software. For analysis, spikes having a mean frequency of 0.2 s were selected, bursts were considered as at least two successive spikes if the minimum interspikes interval was 0.01 s and the minimum burst duration was 0.02 s. For the recording, an automatic threshold estimation, which corresponds to a falling edge of 10, was subtracted.

### PSD fractionation

PSD fractionation was performed as described by Frandemiche et al. ^28^. Briefly, following treatment, primary neurons were recovered in cold lysis buffer containing 10 mM HEPES and 320 mM sucrose, pH 7.4. Homogenates were sonicated and centrifuged at 1000×*g* at 4 °C for 10 min. Supernatants were collected and centrifuged at 12,000×*g* at 4 °C for 20 min. The remaining pellets were washed twice with a buffer containing 4 mM HEPES and 1 mM EDTA, pH 7.4, and centrifuged at 12,000×*g* at 4 °C for 20 min. The remaining pellets were resuspended in a buffer containing 20 mM HEPES, 100 mM NaCl, and 0.5% Triton X-100, pH 7.2. Supernatants were collected as non-PSD fractions. Pellets were resuspended and slowly homogenized for 1 h at 4 °C in a buffer containing 20 mM HEPES, 0.15 mM NaCl, 1% Triton X-100, 1% deoxycholic acid, and 1% SDS, pH 7.5, before centrifugation at 10,000×*g* at 4 °C for 15 min. Supernatants were collected as PSD fractions. Samples were mixed with loading buffer, and equal volumes were loaded and analyzed by Western blotting (WB).

### RT-qPCR

Total RNA was extracted from primary neurons using the NucleoSpin^®^ RNA kit (Macherey–Nagel) following the manufacturer’s instructions. Then, 1 μg of RNA was reverse transcribed using the High Capacity cDNA Reverse Transcription Kit (ThermoFisher Scientific). Quantitative real-time polymerase chain reaction (qRT-PCR) analyses were performed using Power SYBR Green PCR Master Mix (ThermoFisher Scientific) on a StepOne™ Real-Time PCR System (ThermoFisher Scientific) using the following primers: Cyclophilin A forward: 5′-AGCATACAGGTCCTGGCATC-3′, reverse: 5′-TTCACCTTCCCAAAGACCAC-3′; p62 forward: 5′-GCTGCCCTATACCCACATCT-3′, reverse: 5′-CGCCTTCATCCGAAC-3′. PCR reaction conditions were as follow: initial denaturation 95 °C for 10 min, followed by 35 cycles of 95 °C for 15 s, and 60° for 1 min. Fold changes were computed using the ΔΔCt method. ΔCt values were normalized to an internal control Cyclophilin A. Each sample was analyzed in triplicate.

### Statistical analysis

Group comparisons were made using one-way ANOVA with Bonferroni’s post-hoc test or Student’s *t*-test using GraphPad Prism (Prism 5.0d, GraphPad Software Inc., La Jolla, CA, USA). All data are reported as mean ± SD or SEM, and a value of *p* < 0.05 was considered statistically significant.

## Results

### Characterization of primary neuron cultures differentiation

**Fig. 1:**
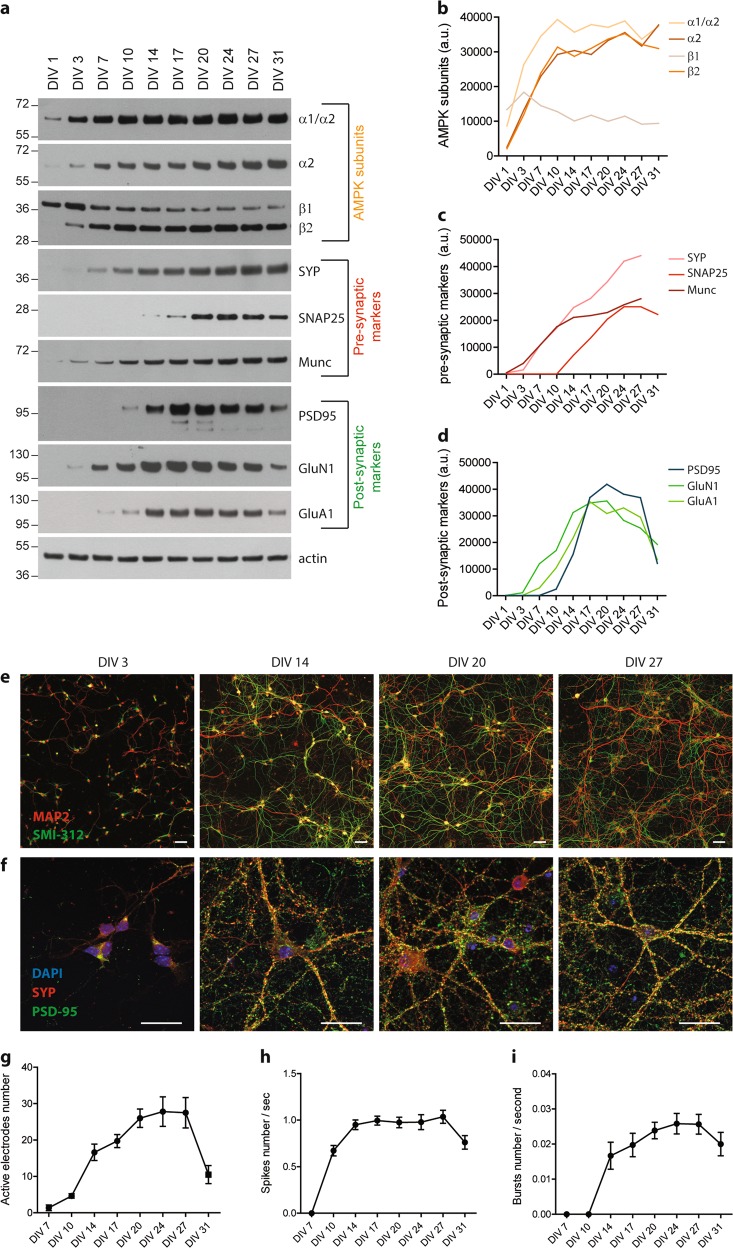
Synaptic markers expression and networks activity during neuronal differentiation. **a–d** Primary neurons were harvested at the indicated days in vitro (DIV). Western blot (WB) analysis (**a**) and quantification of the expression levels of the different AMPK subunits (α1/α2, α2, β1, and β2) (**b**), of the pre-synaptic markers synaptophysin (SYP), SNAP25, and Munc (**c**) and of the post-synaptic markers PSD-95, GluN1 subunit of NMDA receptor, and GluA1 subunit of AMPA receptor (**d**). Results represent mean of four independent experiments; a.u. arbitrary units. **e**, **f** Immunostaining of axons (SMI-312, green) and dendrites (MAP2, red) (**e**) and of the pre-synaptic marker SYP (red) and the post-synaptic marker PSD-95 (green) (**f**) in primary neurons during neuronal differentiation. **g–i** Electrical activity of neuronal networks recorded in primary neurons seeded on multi-electrode arrays (MEA) at the indicated DIV. Quantification of the number of active electrodes (**g**), spikes per second (**h**), and bursts per second (**i**) during neuronal differentiation. Results represent mean ± SEM, *n* = 5–6

To study the role of AMPK hyper-activation on synaptic integrity, we first performed in vitro differentiation kinetics of mouse primary neurons. Primary neurons were maintained up to 31 DIV, and the expression levels of AMPK subunits, pre- and post-synaptic proteins were assessed by WB and immunocytochemistry (ICC). During the first days of differentiation, AMPK subunits expression levels increased to remain constant after 10 DIV (Fig. 1a, b). Regarding synaptic markers, an increased expression of the pre-synaptic proteins SYP, SNAP25, and Munc was observed during the first 17 days of differentiation, which then remained stable up to 31 DIV, except for SNAP25 whose expression decreased after 27 DIV (Fig. 1a, c). The post-synaptic protein PSD-95, the GluN1 subunit of NMDA (*N*-methyl-d-aspartate) receptor, and the GluA1 subunit of AMPA (α-amino-3-hydroxy-5-methyl-4-isoxazolepropionic acid) receptor also rose during neuronal differentiation, reached a plateau at around 17 DIV, and decreased after 27 DIV (Fig. 1a, d).

ICC using the axonal marker SMI-312 and the dendritic marker MAP2 was next performed and showed that during differentiation, cells developed a dense network (Fig. 1e). Co-staining with the pre-synaptic marker SYP and the post-synaptic marker PSD-95 demonstrated the high synaptic density of neuronal networks (Fig. 1f). Finally, the electrical activity of neuronal networks was evaluated using MEA. Neuronal culture on MEA allows a non-invasive and simultaneous recording of electrical activity of whole neuronal networks^29^. Results showed that primary neuron cultures displayed an increasing electrical activity during the differentiation process, defined by an increase in active electrodes, spikes, and bursts numbers (Fig. 1g–i). The maximal electrical activity was reached at 20 DIV and remained stable up to 27 DIV. Neuronal networks activity correlated perfectly with the expression of synaptic markers. Altogether, these data showed that after 20 DIV, neurons had developed extensive networks of dendrites and synapses that were functional and remained healthy for up to 27 DIV.

### AMPK hyper-activation leads to synaptic loss

**Fig. 2:**
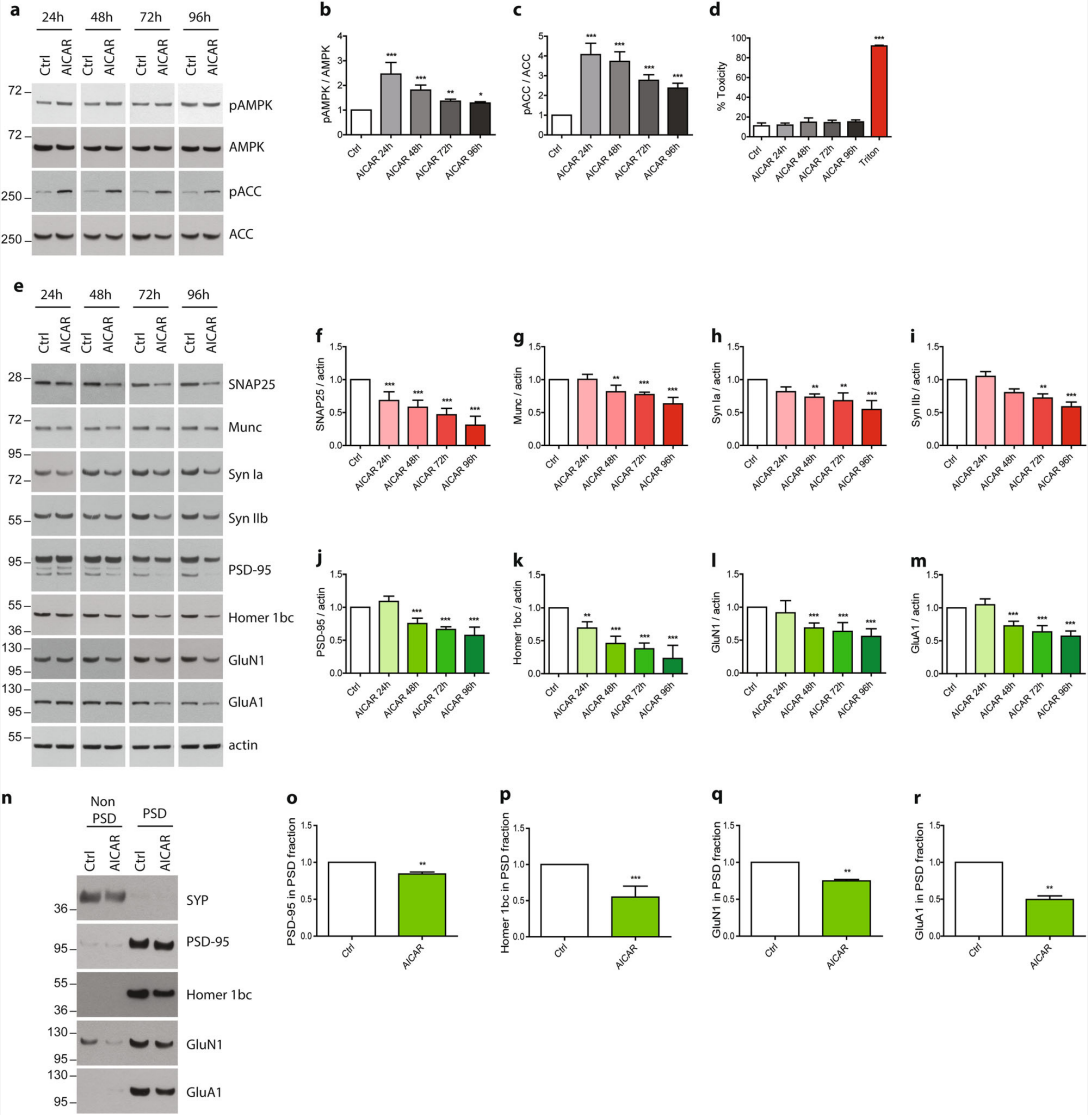
Impact of AMPK hyper-activation on synaptic markers expression. Primary neurons were treated at 20 DIV for the indicated times with the AMPK activator, AICAR (1 mM). **a** Cell lysates were analyzed by WB with antibodies directed against phospho-AMPK-Thr172 (pAMPK), AMPK, phospho-ACC-Ser79 (pACC), ACC, and actin. **b**, **c** Quantification of the ratios pAMPK/AMPK (**b**) and pACC/ACC (**c**). Results represent mean ± SD, *n* = 6. **d** Cytotoxicity was assessed using the lactate dehydrogenase (LDH) assay after 24, 48, 72, and 96 h of AICAR treatment. Treatment with 0.9% Triton X-100 was used as a positive control. Results represent mean ± SD, *n* = 6. **e–m** Synaptic markers expression upon AMPK hyper-activation with AICAR treatment. WB analysis (**e**) and quantification of the pre-synaptic markers SNAP25 (**f**), Munc (**g**), synapsin Ia (Syn Ia) (**h**) and IIb (Syn IIb) (**i**), and the post-synaptic markers PSD-95 (**j**), Homer 1bc (**k**), GluN1 (**l**), and GluA1 (**m**). Results represent mean ± SD, *n* = 6. **n–r** Post-synaptic densities extractions were performed after 48 h AICAR treatment. Resulting non PSD and PSD fraction were analyzed by WB (**n**) and expression of the post-synaptic markers PSD-95 (**o**), Homer 1bc (**p**), GluN1 (**q**), and GluA1 (**r**) was quantified in the PSD fraction. Results represent mean ± SD, *n* = 4. One-way ANOVA with Bonferroni’s post hoc test was used for **b–d** and **f–m** experiments and Student’s *t*-test was used for **o–r** experiments; *p < 0.05, **p < 0.01, ***p < 0.001 compared to Ctrl

To analyze the impact of AMPK hyper-activation on synaptic integrity, AMPK chronic activation was induced at 20 DIV, when neurons were well differentiated in order to avoid any potential effect of AMPK hyper-activation on axon growth as previously described^30,31^. Primary neurons were treated with AICAR (5-aminoimidazole-4-carboxamide ribonucleotide) from 20 up to 24 DIV. AICAR is a cell-permeable nucleoside, which is metabolized by the adenosine kinase in an AMP analog, ZMP (AICA ribotide) that binds to the regulatory subunit of AMPK thus activating it. Samples were collected every day and synaptic markers were assessed by WB and ICC. Upon AICAR treatment, AMPK Thr172 phosphorylation was maintained up to 4 days (Fig. 2a, b) and ACC Ser79 phosphorylation, that was used to monitor AMPK activity, was consistently upregulated at every time point (Fig. 2a, c). WB analysis showed that starting from 48 h of AICAR treatment, a significant reduction of the pre-synaptic proteins SNAP25, Munc, and Synapsin Ia and IIb (Syn Ia, Syn IIb) expression was observed, reaching 40%, 20%, 30%, and 20% of decrease, respectively (Fig. 2e–i). Analysis of the post-synaptic proteins PSD-95 and Homer1 bc expression showed a similar trend, significant reductions were observed starting at 48 h, and reached, respectively, 30% and 50% of decrease as compared to controls (Fig. 2e, j, k). The expression levels of the glutamate receptors subunits GluN1 and GluA1 were also significantly decreased by 30% after 48 h of AICAR treatment (Fig. 2e, l, m). PSD extractions were next performed to determine whether the pool of PSD-localized glutamate receptors was decreased. Following PSD extraction, PSD-95 was exclusively detected in the PSD fraction whereas SYP was recovered in the non-PSD fraction, demonstrating the quality of the extraction protocol^28^. Results demonstrated that the expression of post-synaptically localized glutamate receptors were significantly decreased following AICAR treatment (Fig. 2n–r). Altogether, these data showed that AICAR treatment led to a significant reduction of synaptic markers, and suggested that AMPK hyper-activation might induce a loss of synaptic integrity. In order to rule out the possibility that the decrease of these synaptic markers was due to any cellular toxicity, we performed a cytotoxicity assay. To this end, a quantitative measure of LDH, a cytosolic enzyme which is released upon cell lysis, was realized in the cell media of neurons treated or not with AICAR up to 96 h (Fig. 2d). Additionally, a cell stress array was performed to monitor the expression or activation of 26 cell stress-related proteins. Results demonstrated that no significant toxicity or induction of cell stress were detected (Fig. 2d and Fig. S1). Altogether, these results showed that pharmacological AMPK hyper-activation led to a reduction of synaptic proteins without inducing any toxicity or apparent cellular stress during the first 72 h of treatment.

**Fig. 3:**
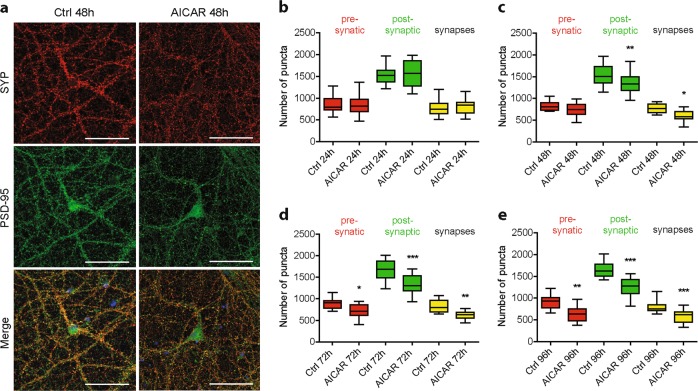
Effect of AMPK hyper-activation on synaptic density. AMPK hyper-activation was induced in primary neurons using AICAR (1  mM) at 20 DIV for the indicated times. **a** Immunostaining using antibodies directed against the pre-synaptic marker synaptophysin (SYP, red) and the post-synaptic marker PSD-95 (green) on neurons treated or not with AICAR for 48 h. **b–e** Quantification of pre-synaptic (red), post-synaptic (green), and synaptic (yellow) puncta upon AICAR treatment for 24 h (**b**), 48 h (**c**), 72 h (**d**), and 96 h (**e**). Results represent mean ± SEM, *n* = 32 neurons counted from three independent experiments. One-way ANOVA with Bonferroni’s post hoc test; *p < 0.05, **p < 0.01, ***p < 0.001. Scale bar = 50 μm

To go further, ICC was implemented using the pre-synaptic marker SYP and the post-synaptic marker PSD-95 to allow quantification of synapses number (Fig. 3a). To this end, puncta analysis using the synaptcountJ plugin for ImageJ was performed as previously described^27,32^. Pharmacological AMPK hyper-activation using AICAR showed that post-synaptic puncta were significantly reduced after 48 h of treatment (1533 puncta ± 52 in control condition compared to 1342 puncta ± 59 upon AICAR treatment), while pre-synaptic puncta were significantly reduced starting at 72 h (894 puncta ± 35 in control condition compared to 714 puncta ± 33 in AICAR condition). The decrease in post-synaptic and pre-synaptic puncta correlated with a significant reduction of synaptic puncta (767 puncta ± 23 in control condition compared to 603 puncta ± 24 upon AICAR treatment), that corresponded to the co-distribution of both pre- and post-synaptic markers (Fig. 3a–e). Overall, these results correlated with the decreased expression of synaptic proteins detected by WB and demonstrated that AMPK hyper-activation led to a loss of synapses.

To further demonstrate the implication of AMPK, similar experiments were performed using a direct AMPK activator, GSK621^33,34^. We first determined the efficiency of this drug by analyzing by WB the phosphorylation levels of AMPK Thr172 and ACC Ser79 and showed that GSK621 induced a significant activation of AMPK up to 96 h of treatment (Fig. S2a-c) without inducing any toxicity (Fig. S2d). Similar to AICAR, we found that GSK621 treatment for 48 h led to a significant reduction of the expression of the pre-synaptic proteins SNAP25 and synapsin Ia by 60% and 40%, respectively (Fig. S2e-g). The expression of the post-synaptic proteins PSD-95, Homer 1bc, and the glutamate receptor subunit GluN1 were also significantly decreased by 50%, 50%, and 30%, respectively (Fig. S2e, h-j). Additionally, quantification of synapses number showed that after 48 h of treatment with GSK621, the number of pre-synaptic puncta were significantly reduced (860 puncta ± 29 in control conditions compared to 679 puncta ± 24 upon GSK621 treatment), as well as the number of post-synaptic puncta (1362 puncta ± 64 in control conditions compared to 1105 puncta ± 63 upon GSK621 treatment), and therefore the number of synaptic puncta (723 puncta ± 35 in control conditions compared to 514 puncta ± 34 upon GSK621 treatment) (Fig. S2k-o). Overall, these results confirm those obtained with AICAR showing that AMPK hyper-activation induced synaptic loss.

### AMPK-mediated synaptic loss results in neuronal networks activity impairment

**Fig. 4:**
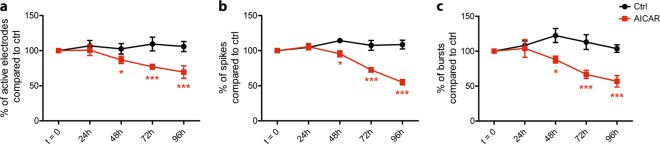
Effect of AMPK hyper-activation on neuronal networks activity. Primary neurons seeded on MEA were subjected or not to AMPK hyper-activation with AICAR (1 mM) at 20 DIV. **a–c** Electrical activity was recorded for the indicated times. Quantification of the number of active electrodes (**a**), spikes (**b**), and bursts (**c**) expressed in percentage of *t* = 0. Results represent mean ± SEM, *n* = 5–6. One-way ANOVA with Bonferroni’s post hoc test; *p < 0.05, ***p < 0.001 compared to *t* = 0

To determine whether the synaptic loss mediated by AMPK hyper-activation had an impact on synapses functionality, we assessed the electrical activity of neuronal networks using MEA. Primary neurons were cultured on MEA, and AMPK hyper-activation was induced pharmacologically using AICAR. Neuronal networks activity was recorded before treatment and every day for 96 h. Results showed that the number of active electrodes was significantly reduced by 16% following 48 h of AICAR treatment (Fig. 4a). In parallel, the number of spikes and bursts, which represent at least 2 consecutive spikes, were also significantly reduced by 19% and 34%, respectively, after the treatment (Fig. 4b, c). Altogether, these results demonstrated that besides inducing a reduction in synaptic proteins expression, AMPK hyper-activation led to an impairment of neuronal networks activity that correlated with the decrease in synapses number.

### AMPK induces synaptic loss through autophagy-mediated clearance

**Fig. 5:**
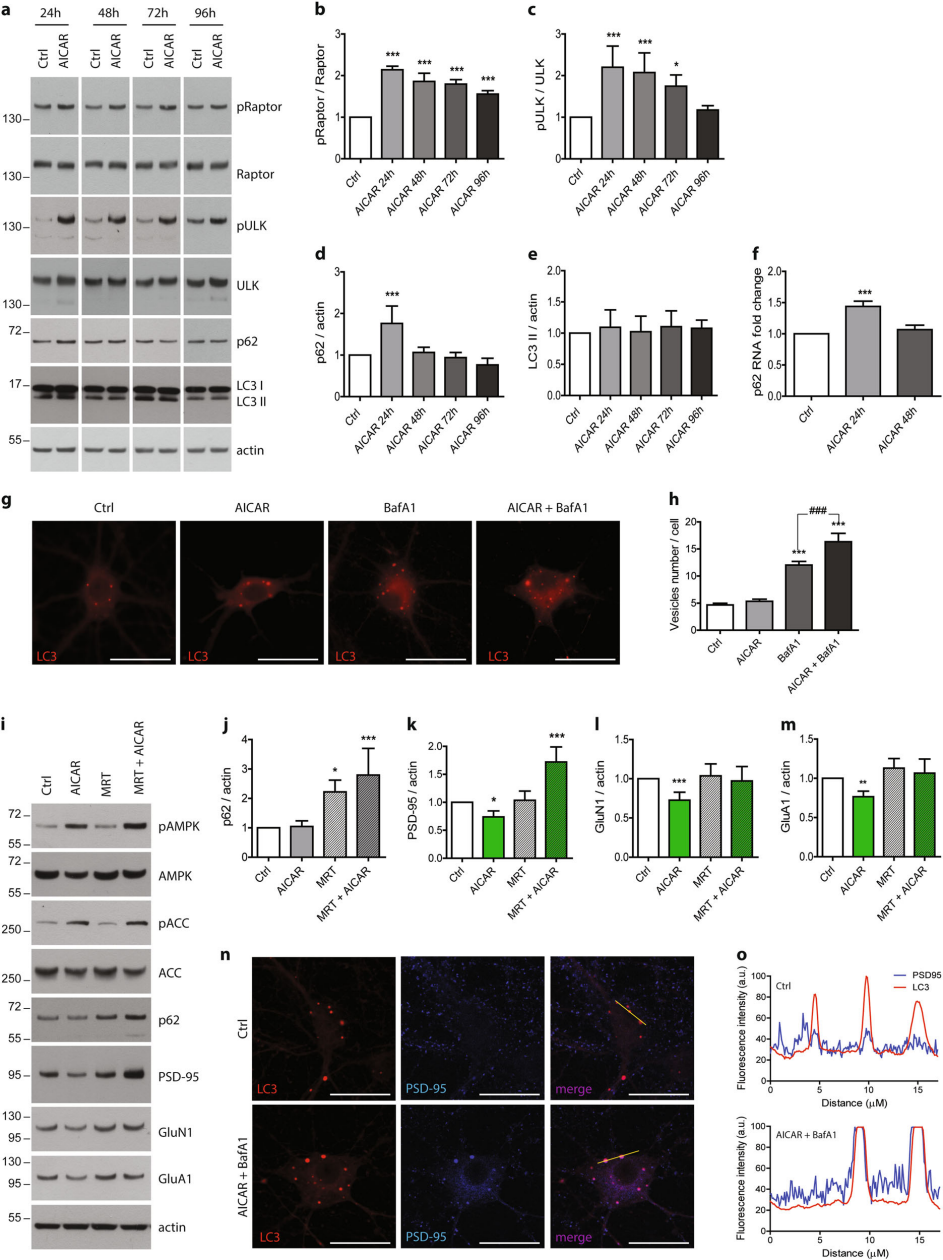
Involvement of autophagy in post-synaptic markers degradation. Neurons were treated with AICAR (1 mM) at 20 DIV for the indicated times. **a** Cell lysates were analyzed by WB for phospho-Raptor-Ser792 (pRaptor), Raptor, phospho-ULK-Ser555 (pULK), ULK1, and p62. **b–e** Quantification of the ratios pRaptor/Raptor (**b**), pULK/ULK (**c**), p62/actin (**d**), and LC3 II/actin (**e**). Results represent mean ± SD, *n* = 4. **f** p62 mRNA levels assessed by RT-qPCR after 24 h and 48 h of AICAR treatment. Results represent mean ± SD, *n* = 7. **g** Visualization of mKate2-LC3 in neurons treated with AICAR (1 mM, 48 h), BafilomycinA1 (BafA1, 100 nM, 24 h), or both. **h** Quantification of mKate2-LC3 positive vesicles number by cell. Results represent mean ± SEM, *n* = 30 neurons counted from three independent experiments. Scale bar = 25 µm. **i–m** Primary neurons at 20 DIV were pre-treated with the autophagy inhibitor MRT68921 (MRT, 2.5 µM) for 20 min prior to AICAR treatment (1 mM, 48 h). WB analysis (**i**) and quantification of p62 (**j**), PSD-95 (**k**), GluN1 (**l**), and GluA1 (**m**). Results represent mean ± SD, *n* = 4. **n** PSD-95 immunostaining (blue) in neurons expressing mKate2-LC3 (red) and co-treated with AICAR (1 mM, 48 h) and BafilomycinA1 (BafA1, 100 nM, 24 h). **o** Fluorescence profile corresponding to the yellow lines in **n** and showing the co-distribution of LC3 and PSD-95 in neurons co-treated with AICAR and BafA1. Scale bar = 25 µm; a.u. arbitrary units. One-way ANOVA with Bonferroni’s post hoc test was used for all experiments; *p < 0.05, **p < 0.01, ***p < 0.001 compared to the Ctrl, ^###^*p* < 0.001 compared to BafA1 condition

Finally, we wanted to determine the mechanism by which the synaptic loss occurred. As mentioned previously, AMPK regulates the autophagic pathway by directly phosphorylating the autophagy initiator ULK1 at Ser317, 555, 777^14^and by indirectly impairing mTORC1-dependent inhibition of ULK1 through the direct phosphorylation of the protein Raptor at Ser792. Therefore, we hypothesized that autophagy might be implicated in AMPK-mediated synaptic loss. We first determined whether autophagy markers were changed following AMPK hyper-activation by assessing the phosphorylation levels of ULK1 and Raptor. We observed an increase in ULK1 and Raptor phosphorylation at Ser555 and Ser792, respectively, following AICAR or GSK621 treatment during 72 h for ULK1 and at all time points studied for Raptor (Fig. 5a–c, and Fig. S3a). Next, we evaluated the levels of SQSTM1/p62, an ubiquitin-binding protein degraded in autolysosomes, and the lipidation of the microtubule-associated protein 1 light chain 3 (LC3) to assess the autophagic flux following AMPK activation. p62 levels were significantly increased following 24 h of AICAR treatment (Fig. 5a, d). p62 increase could either result from an inhibition of autophagic vesicles clearance or from transcriptional upregulation. To address the latter possibility, *SQSTM1/p62* mRNA levels were monitored by qRT-PCR following AICAR. Results showed that *SQSTM1/p62* mRNA levels were significantly increased after 24 h of treatment (Fig. 5f), suggesting that the increase in SQSTM1/p62 levels likely resulted from transcriptional upregulation. Likewise, LC3 levels and lipidation were not affected by AICAR treatment (Fig.5 a, e), further supporting an increased autophagic activity following AMPK activation. Finally, lysosomal degradation of autophagosomes was prevented by treating neurons with Bafilomycin A1 (BafA1), an inhibitor of the vacuolar H^+^ ATPase known to inhibit autolysosomes acidification and autophagosome–lysosome fusion, to confirm autophagic flux activation. Quantification of autophagosomes in LC3 (pHluorin-mKate2) expressing neurons showed that significantly more autophagosomes accumulated when neurons were co-treated with AICAR or GSK621 and BafA1 as compared to the BafA1 condition (Fig. 5g, h and Fig. S3b, c), thus clearly demonstrating that the autophagic flux was stimulated following AMPK hyper-activation. These data are in line with a recent study showing that AMPK is involved in the autophagic flux by upregulating autophagosomes maturation and autolysosomes fusion^35^.

Next, to determine whether autophagy induction was involved in the synaptic loss induced by AMPK hyper-activation, primary neurons were pre-treated with the specific ULK1 inhibitor MRT68921 20 min before AMPK stimulation with AICAR or GSK621. Autophagy initiation blockade was evidenced by the significant increase in p62 protein levels following MRT68921 treatment (Fig. 5i, j and Fig. S3d). After 48 h of AMPK stimulation, we found that the inhibition of autophagy initiation reversed the effects of AMPK activation on the synaptic proteins PSD-95, GluN1, and GluA1 (Fig. 5i, k–m and Fig. S3d, f, g). Finally, ICC using an antibody directed against PSD-95 in neurons co-treated with AICAR or GSK621 and BafA1 showed the co-distribution of PSD-95 and LC3-positive vesicles (Fig. 5n, o and Fig. S4h, i), further supporting the finding that post-synaptic markers degradation following AMPK hyper-activation is mediated by autophagy. However, the blockage of autophagy by MRT68921 did not reverse the decrease in pre-synaptic proteins expression, nor pre-synaptic or synapses puncta induced by AMPK hyper-activation (Fig. S4a-e). Altogether, these data seem to indicate that post-synaptic proteins are more susceptible to AMPK and autophagy activation than pre-synaptic proteins. Interestingly, the distribution of the AMPK subunits in PSD fractions indicates that all of the AMPK subunits studied are recovered in the enriched post-synaptic fraction (Fig. S4f), which may explain why post-synaptic proteins are the most vulnerable to AMPK hyper-activation.

Overall, these results showed that ULK1-induced autophagy was involved at least in the degradation of post-synaptic proteins mediated by AMPK hyper-activation.

### In vivo hyper-activation of AMPK leads to synaptic loss

**Fig. 6:**
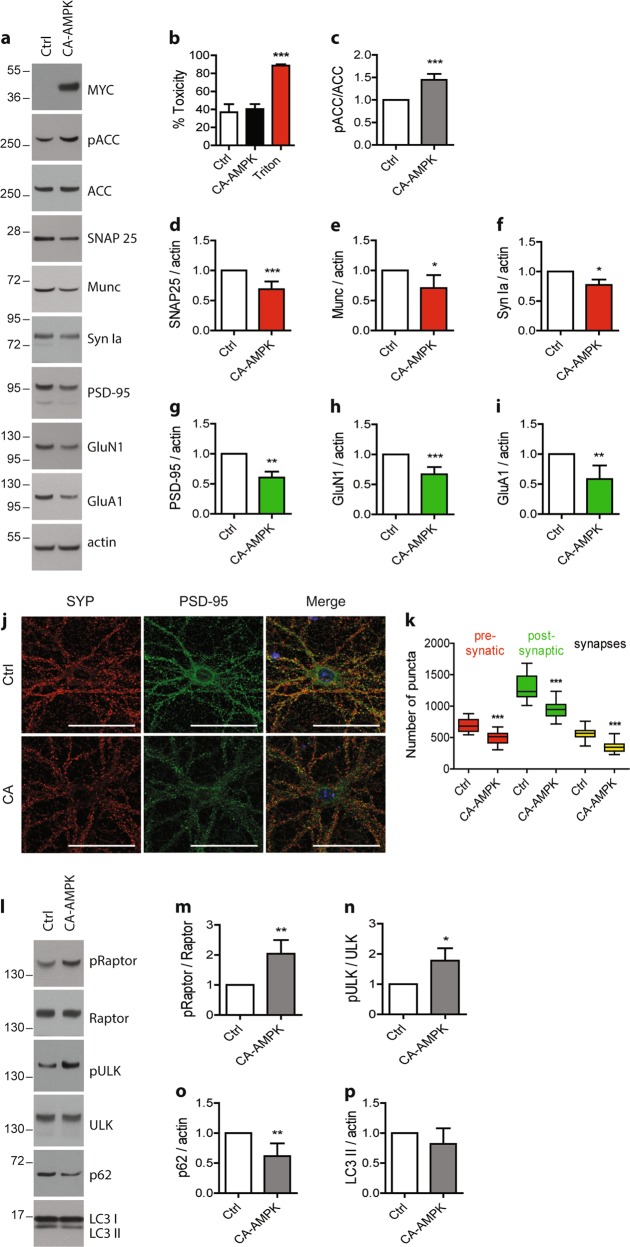
Effect of CA-AMPK expression on synaptic integrity, density, and autophagic markers. Primary neurons were infected at 17 DIV with a constitutively active form of AMPK (CA-AMPK) for 7 days. **b** Cytotoxicity was assessed by measuring the lactate dehydrogenase (LDH) released in cell media. Treatment with 0.9% Triton X-100 was used as a positive control. Results represent mean ± SD, *n* = 4. Cells lysates were analyzed by WB (**a**) and the ratio pACC/ACC was quantified (**c**). **d–i** Quantification of the pre-synaptic markers SNAP25 (**d**), Munc (**e**), and synapsin Ia (**f**) and the post-synaptic markers PSD-95 (**g**), GluN1 (**h**), and GluA1 (**i**). Results represent mean ± SD, *n* = 8. **j** Immunostaining of synaptophysin (SYP, red) and PSD-95 (green) in primary neurons infected at 17 DIV with the CA-AMPK for 7 days. **k** Quantification of pre-synaptic (red), post-synaptic (green), and synaptic (yellow) puncta upon CA-AMPK expression. Results represent mean ± SEM, *n* = 38 neurons counted from five independent experiments. Scale bar = 50 µm. **l–p** Analysis of the expression of proteins involved in autophagy pathway. Quantification of the ratios pRaptor/Raptor (**m**) and pULK/ULK (**n**) and of the expression level of p62 (**o**) and LC3 II proteins (**p**). Results represent mean ± SD, *n* = 6. Student’s *t*-test was performed for **c–i** and **m–p** experiments. One-way ANOVA with Bonferroni’s post hoc test was used for **b**, **k** experiments; *p < 0.05, **p < 0.01, ***p < 0.001 compared to Ctrl

To assess the consequences of AMPK hyper-activation in vivo, we took advantage of a constitutively active AMPK construct (CA-AMPK). We first determined the consequences of overexpressing CA-AMPK in vitro in primary neurons on synaptic integrity. To this end, primary neurons were infected with LV to deliver the CA-AMPK at 17 DIV up to 24 DIV. Efficiency of the CA-AMPK was confirmed by assessing the phosphorylation level of ACC Ser79 after 7 days of transduction (Fig. 6a, c). Then, we analyzed the expression of the pre- and post-synaptic proteins. Upon CA-AMPK expression, we observed a significant reduction of the expression of the pre-synaptic proteins SNAP25, Munc, and synapsin Ia by 30%, 30%, and 20%, respectively (Fig. 6a, d–f) and of the post-synaptic proteins PSD-95, GluN1, and GluA1 by 40%, 35%, and 40%, respectively (Fig. 6a, g–i), which were not associated to any cell toxicity (Fig. 6b). Quantification of synapses demonstrated that following CA-AMPK expression, the number of synapses was significantly decreased (puncta 575 ± 18 in control conditions compared to puncta 350 ± 13 upon CA infection) (Fig. 6j, k). CA-AMPK overexpression also led to an activation of the autophagic pathway. Indeed, upon CA-AMPK expression, we observed a significant increase in Raptor phosphorylation level on Ser792 and ULK1 on Ser555 while p62 protein expression was significantly decreased (Fig. 6l–o). Moreover, the expression level of LC3 II was not impacted by the expression of the CA-AMPK (Fig. 6l, p). Altogether, these data demonstrated that CA-AMPK displayed the same effect on synaptic integrity through the regulation of autophagy than the pharmacological hyper-activation of AMPK.

**Fig. 7:**
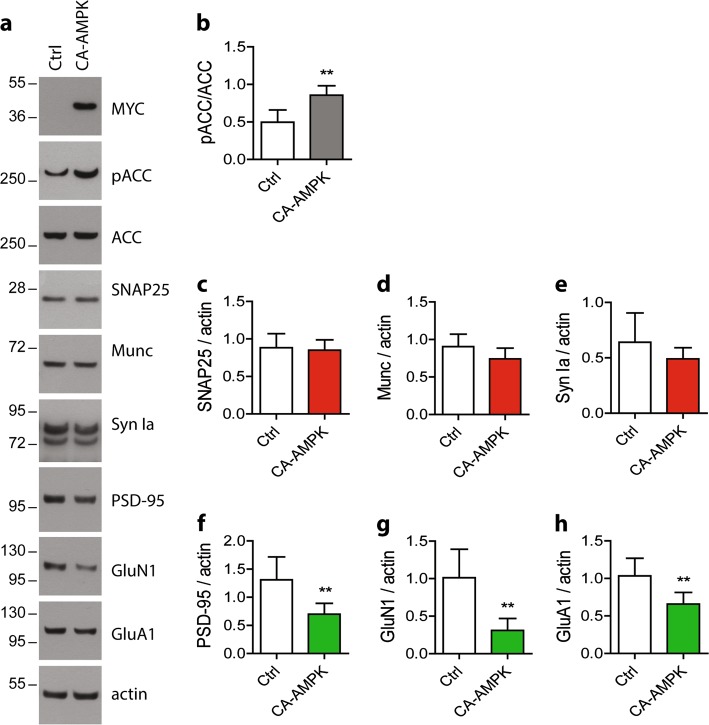
Impact of AMPK hyper-activation on synaptic markers in vivo in mice. Two-month-old WT mice were injected with lentiviral vectors (LV) to allow the expression of the CA-AMPK in the hippocampus. Hippocampus were extracted 1 month after LV injections and lysates were analyzed by WB (**a**). WB quantification of pACC/ACC ratio (**b**) and of the expression level of SNAP 25 (**c**), Munc (**d**), Synapsin Ia (**e**), PSD-95 (**f**), GluN1 (**g**), and GluA1 (**h**) in the hippocampus of CA-AMPK or Ctrl mice. Results represent mean ± SD, *n* = 6 (Ctrl) and *n* = 8 (CA-AMPK). Student’s *t*-test; **p < 0.01, compared to Ctrl

To further determine whether AMPK could also be involved in the regulation of synaptic integrity in vivo, CA-AMPK was expressed in the dorsal hippocampus of wild-type mice. A month after the injection of LV for the delivery of CA-AMPK, hippocampus were extracted and WB were performed. Our results showed that in the CA-AMPK group, the post-synaptic proteins PSD-95, GluN1, and GluA1 were significantly reduced as compared to the Ctrl group (Fig. 7a, f–h). While non-significant, we also observed a decreasing trend for the pre-synaptic proteins SNAP25, Munc, and Synapsin Ia (Fig. 7a, c–e).

Altogether, these data demonstrated that AMPK hyper-activation in vivo in the mouse brain also led to a decrease in synaptic integrity.

## Discussion

Here, we report that AMPK hyper-activation, as it is observed in AD, led to a loss of synaptic markers in cultured primary neurons. This decrease in synaptic markers was associated with a loss of synapses and accompanied by a decrease in neuronal networks functionality. Additionally, we found that autophagy was involved in the degradation of synaptic markers. Finally, we confirmed that AMPK hyper-activation also induced a loss of synaptic markers in vivo in the mouse hippocampus. These results are in line with a previous study showing that AMPK-mediated dendritic spine loss induced by β-amyloid oligomers in hippocampal primary neurons and in vivo in the APP^SWE,IND^ transgenic mouse model^23^.

While physiological synapse elimination, known as synaptic pruning, occurs during adolescence in humans, synaptic loss in adults is associated with cognitive impairments and is an early hallmark of neurodegenerative diseases including Alzheimer’s^3^. However, the exact cellular and molecular mechanisms that are responsible for synaptic loss remain poorly understood. Nonetheless, synaptic proteins elimination could be mediated through autophagy-dependent processes. For instance, AMPA receptors are degraded through autophagy after chemical long-term depression^36^. In addition, proper regulation of autophagy is necessary to preserve neuronal integrity. In particular, downregulation of Atg7, an enzyme required for autophagosome formation, significantly reduces spines elimination in primary neurons^37^. Further studies showed that Atg5 or Atg7 knockout in animal models leads to neurodegeneration^38,39^. Here, we extend on these previous findings and demonstrate that post-synaptic proteins elimination, at least for PSD-95, GluN1, and GluA1, is mediated by an AMPK-driven autophagy-dependent process. However, how these post-synaptic components are targeted for lysosomal degradation remains to be established. It is possible that ubiquitination could be involved. Indeed, selective autophagy is a process that degrades K63-linked polyubiquitinated chained or monoubiquitinated substrates^40,41^. In this process, the polyubiquitin chain is recognized by adaptor proteins, such as p62, which act as bridges to link ubiquitin to autophagosomes^42^. Interestingly, PSD-95 was reported to undergo K63 polyubiquitination^43^, and the ubiquitination of GluA1 in response to AMPA was reported to mediate the endocytosis and lysosomal trafficking of GluA1-containing AMPA receptors^44^. Intriguingly, our results suggest that pre-synaptic proteins (SNAP25, synapsin, and Munc) are eliminated following AMPK hyper-activation by a process that is not dependent on ULK1-regulated autophagy. Indeed, ULK1 inhibition could not reverse the loss of these pre-synaptic proteins in our model. However, it is worth noting that ULK1-independent autophagy can also occur^45^. Therefore, we cannot exclude the possibility that pre-synaptic autophagy occurs and might be involved in the elimination of these pre-synaptic proteins or of other pre-synaptic components, including synaptic vesicles. Further investigations will be required to address this question and to determine the mechanism by which SNAP25, synapsin, and Munc are eliminated following AMPK hyper-activation.

Autophagy-mediated elimination of post-synaptic proteins also suggests that autophagy could play a role in synaptic plasticity. Interestingly, few studies have assessed the role of autophagy in synaptic functions, and whether synaptic plasticity is affected by autophagic flux remains to be clearly established. Nonetheless, some interesting studies have shown a role for autophagy in pre-synaptic buttons. Autophagy is locally regulated in the pre-synaptic compartment by the scaffolding proteins of the pre-synaptic zone, Bassoon and Piccolo^46^. Pre-synaptic autophagy regulates synaptic vesicles elimination in motorneurons^47^ and dopaminergic axons^39^, thus regulating neurotransmitter release. Autophagic degradation of post-synaptic proteins was also proposed to be involved in BDNF-induced synaptic plasticity^48^. Interestingly, a recent study demonstrated that autophagy is required to erase auditory fear memory through synaptic destabilization^49^. In our study, we found that hyper-activation of AMPK induced autophagic clearance of post-synaptic components that correlated with a loss of neuronal networks activity. Altogether, these results suggest that AMPK hyper-activation could impair synaptic plasticity and memory. Interestingly, pharmacological AMPK activation with AICAR or metformin was reported to impair synaptic plasticity in ex vivo hippocampal slices through a mechanism involving mTOR signaling^50^. Similarly, AMPK hyper-activation is also responsible for the synaptic defects mediated by β-amyloid peptides^51^. Overall, these studies showed that AMPK hyper-activation has a detrimental effect on synaptic plasticity and therefore could impact memory formation. In this context, previous studies have shown that intrahippocampal infusion of AICAR in WT rats impaired their spatial memory^52^, and that expression of the CA-AMPK construct in the CA1 region of rat hippocampus repressed the formation of long-term fear memory^53^. However, further work will be necessary to clearly establish the exact role of the AMPK-regulated autophagy pathway on these processes.

Beside its role in clearing misfolded or aggregated proteins and damaged organelles, autophagy is important for balancing energy sources in response to nutrient stress to allow cells to reutilize their own constituents^54^. Importantly, synapses, and in particular post-synaptic compartments, are the major site of energy consumption in the brain. Overall, our data suggest that AMPK hyper-activation through the enhancement of synaptic constituents degradation via autophagy could help preserve neuronal energy. In addition, the elimination of functional synapses by reducing neuronal energy consumption could promote neuronal survival in conditions of energy stress. It is interesting to note that AMPK was previously reported in other cell models to promote cellular senescence, an adaptation mechanism that allows cellular survival. For instance, increased AMPK activity is found in senescent fibroblasts;^55,56^ and LKB1, the main AMPK kinase, is upregulated in endothelial senescent cells and its overexpression can induce senescence through AMPK activation^57^. While senescence has long been attributed to dividing cells, evidences also suggest that a senescence-like phenotype can be observed in post-mitotic neurons^58^. Very interestingly, two recent papers investigated the role of cellular senescence in neurodegeneration and showed that the elimination of senescent neurons or glial cells using genetic tools or senolytics prevented or slowed neurodegeneration and cognitive decline in mouse models of tauopathies^59,60^. It would be interesting in this context, to determine whether AMPK hyper-activation could induce a senescence-like phenotype in neurons.

## Conclusions

Here, we demonstrate that hyper-activation of the metabolic sensor and regulator AMPK is sufficient to induce synaptic loss and neuronal networks dysfunction. Our data also show that autophagy is induced in response to AMPK hyper-activation and participates in the elimination of post-synaptic proteins.

Altogether, our work has established AMPK as a potential link between energetic failure and synaptic integrity in neurodegenerative conditions such as AD, and it suggests that autophagy might link environmental conditions with synaptic plasticity through the AMPK pathway.

## Supplementary information

Supplementary Figure 1

Supplementary Figure 2

Supplementary Figure 3

Supplementary Figure 4

Supplementary Figure legend
