# Supplementary figures and images for: Neuronal AMP-activated protein kinase hyper-activation induces synaptic loss by an autophagy-mediated process
Source: Cell Death Dis. 2019 Mar 4;10(3):221. doi: 10.1038/s41419-019-1464-x (PMC6399353; doi:10.1038/s41419-019-1464-x)

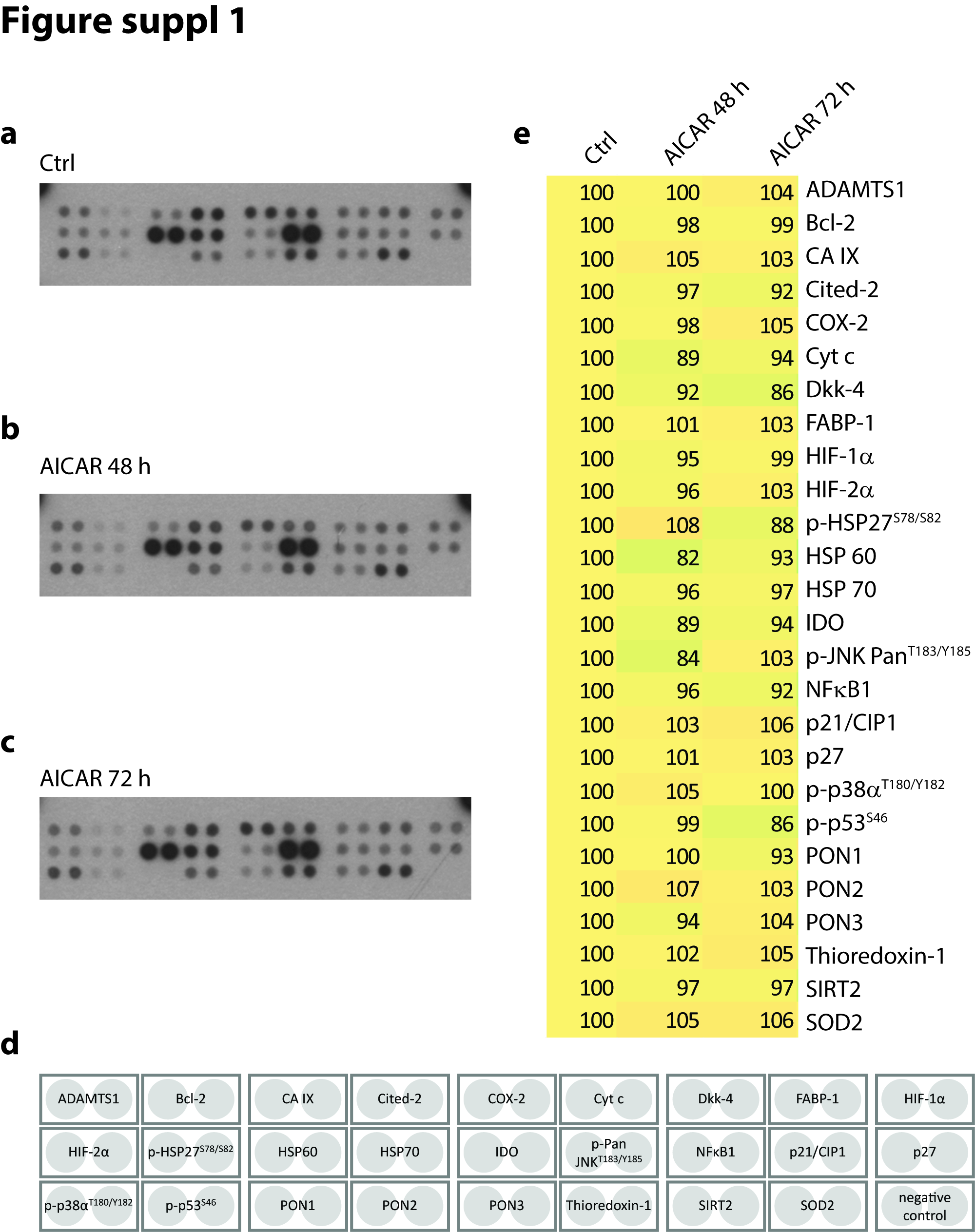

Supplement: Supplementary file 1 — Supplementary Figure 1 [file 41419_2019_1464_MOESM1_ESM.tif]

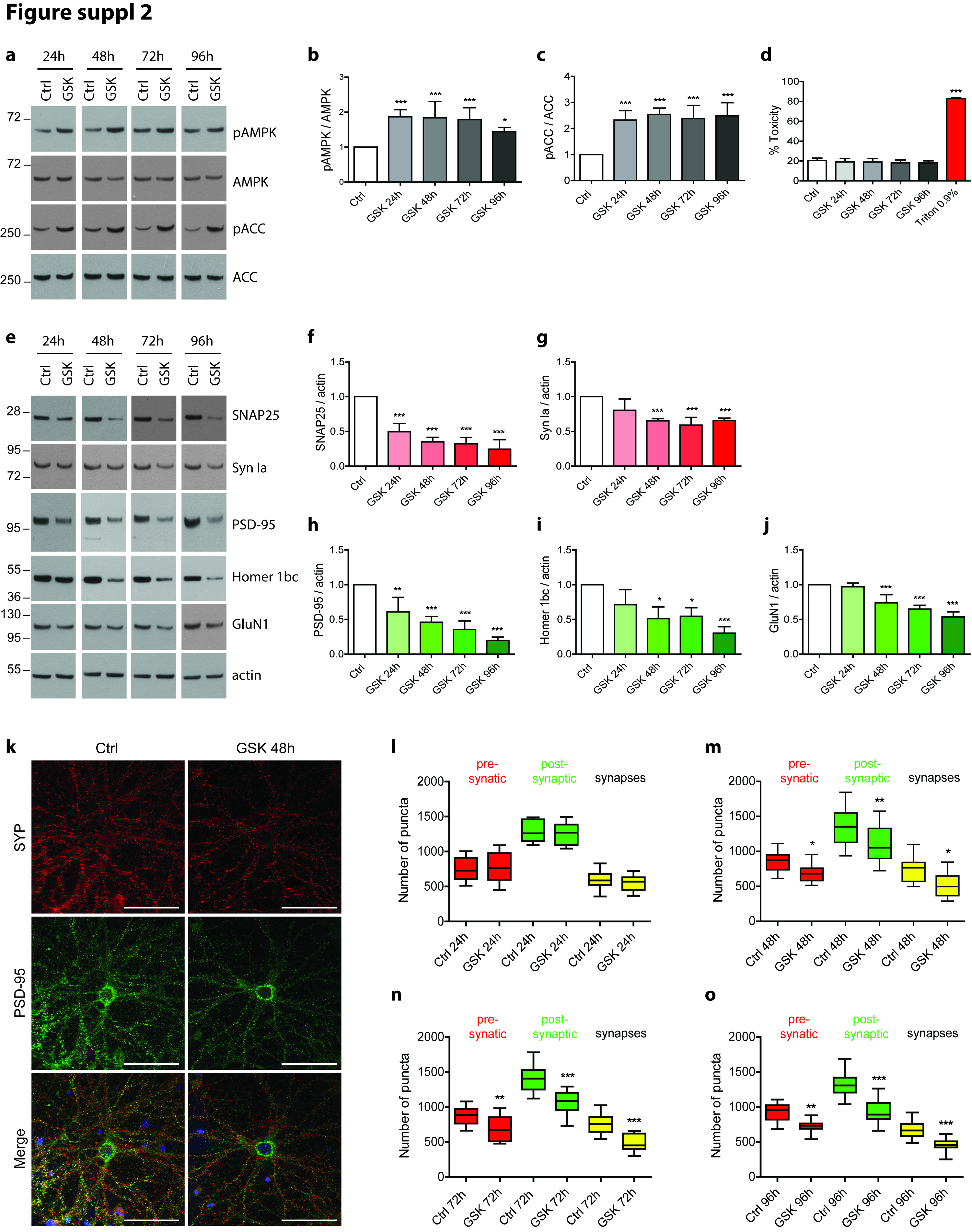

Supplement: Supplementary file 2 — Supplementary Figure 2 [file 41419_2019_1464_MOESM2_ESM.tif]

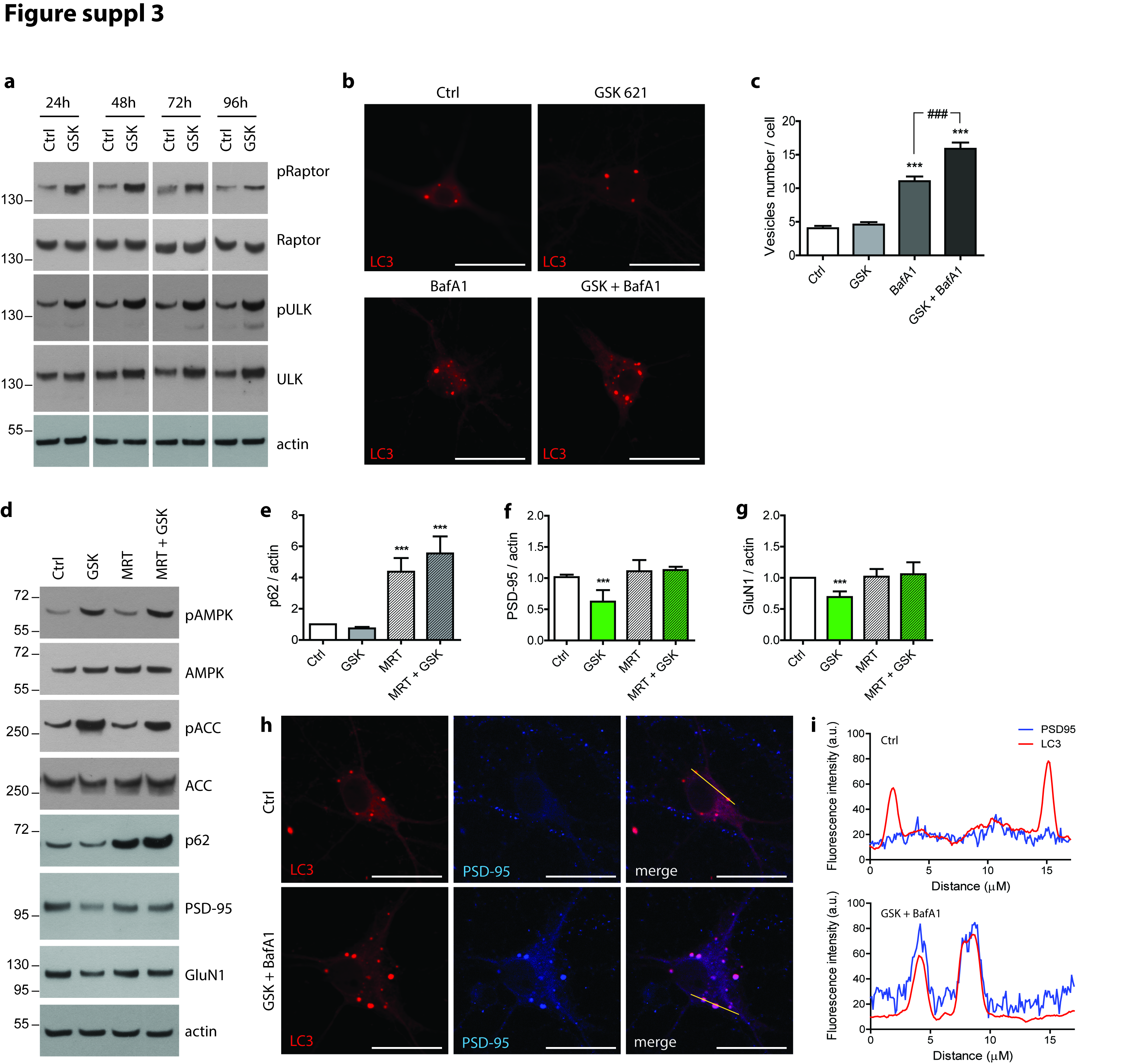

Supplement: Supplementary file 3 — Supplementary Figure 3 [file 41419_2019_1464_MOESM3_ESM.tif]

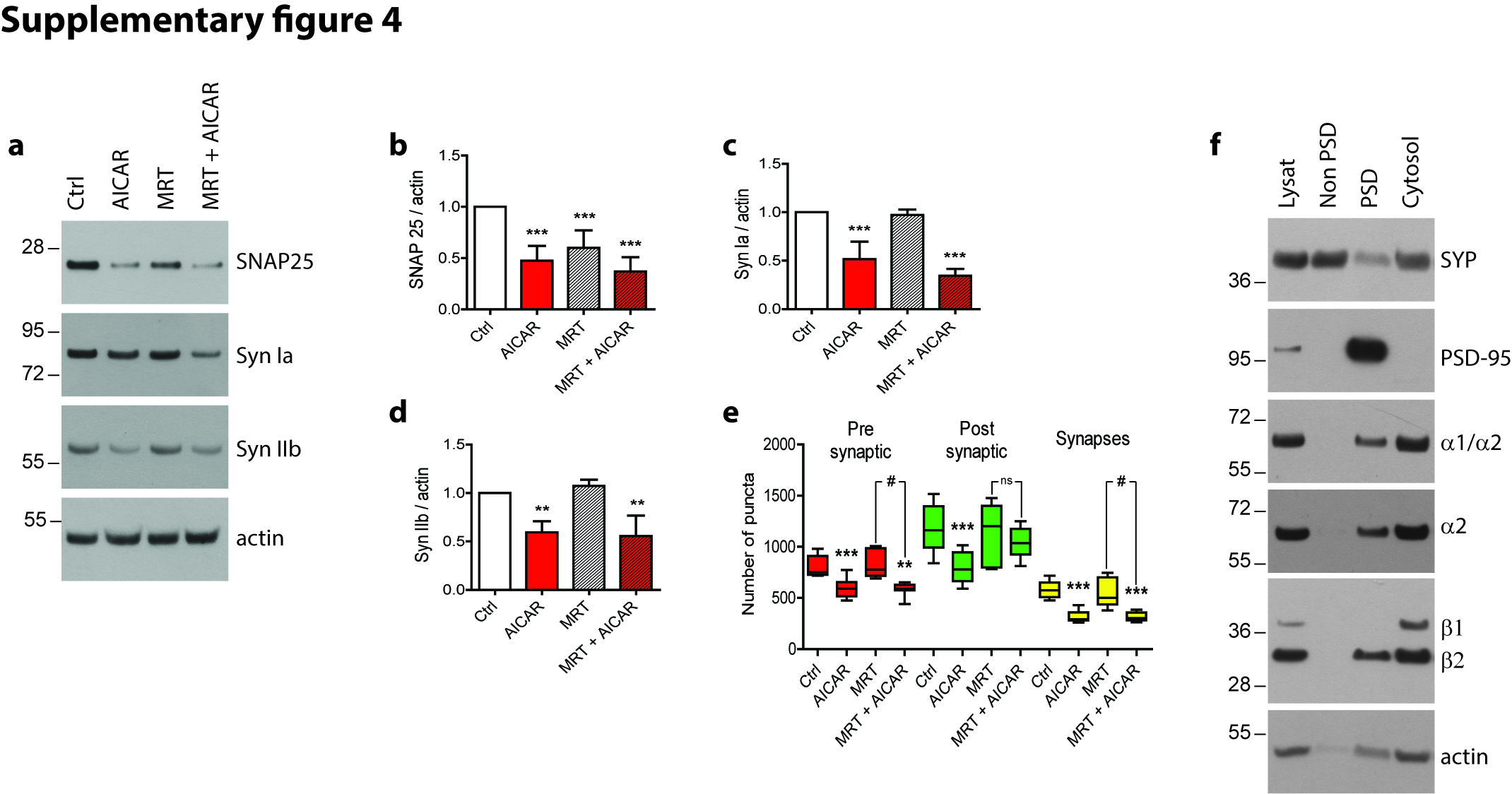

Supplement: Supplementary file 4 — Supplementary Figure 4 [file 41419_2019_1464_MOESM4_ESM.tif]
