# Supplementary material for: Neuronal AMP-activated protein kinase hyper-activation induces synaptic loss by an autophagy-mediated process
Source: Cell Death Dis. 2019 Mar 4;10(3):221. doi: 10.1038/s41419-019-1464-x (PMC6399353; doi:10.1038/s41419-019-1464-x)
Supplement: Supplementary file 5 — Supplementary Figure legend [file 41419_2019_1464_MOESM5_ESM.docx]

**Additional** Fig. 1: **AMPK hyper-activation-induced loss of synaptic markers was not mediated by cell stress.**

(**a-c**) Lysates from differentiated primary neurons treated with AICAR (1▒mM) at DIV 20 for the indicated times were subjected to a cell stress array. (**d**) Schematic representing the localization of stress proteins on the membrane-based antibody array. (**e**) Quantification of the cell stress array represented as a heat map, results are expressed as percentage of the Ctrl.

**Additional Fig. 2: Impact of AMPK hyper-activation on synaptic integrity using the direct AMPK activator GSK621**

Primary neurons were treated at 20 DIV for the indicated times with the direct AMPK activator, GSK621 (GSK, 10▒µM). (**a**) Cells lysates were analyzed by WB with antibodies directed against phospho-Thr^172^AMPK (pAMPK), AMPK, phospho-Ser^79^ACC (pACC), ACC and actin. (**b-d**) Quantification of the ratios pAMPK/AMPK (**b**) and pACC/ACC (**c**). Results represent mean▒±▒SD, n▒=▒5. (**d**) Cytotoxicity was assessed using the lactate dehydrogenase (LDH) assay after 24▒h, 48▒h, 72▒h and 96▒h of treatment with GSK621 in conditioned media. Treatment with 0.9% Triton X-100 was used as a positive control. Results represent mean▒±▒SD, n▒=▒5. (**e-j**) Analysis of synaptic markers upon AMPK hyper-activation with GSK621 treatment. WB analysis (**e**) and quantification of the expression of the pre-synaptic markers SNAP25 (**f**) and synapsin Ia (**g**) and the post-synaptic markers PSD-95 (**h**), Homer 1bc (**i**) and GluN1 (**j**). Results represent mean▒±▒SD, n▒=▒6 (**k**) Immunofluorescence performed with antibodies directed against the pre-synaptic marker synaptophysin (SYP, red) and the post-synaptic marker PSD-95 (green) in neurons treated or not with GSK621 for 48▒h. (**l-o**) Quantification of pre-synaptic (red), post-synaptic (green) and synaptic (yellow) puncta upon GSK621 treatment for 24▒h (**l**), 48▒h (**m**), 72▒h (**n**) and 96▒h (**o**). Results represent mean▒±▒SD, n▒=▒31 neurons counted from 3 independent experiments. Scale bar▒=▒50 μm.

One way ANOVA with Bonferroni’s post hoc test was performed for all experiments. *p▒<▒0.05, **p▒<▒0.01, ***p▒<▒0.001.

**Additional Fig. 3: Involvement of autophagy on synaptic loss induced by GSK621**

Neurons were treated at 20 DIV for the indicated times with GSK621 (GSK, 10▒µM). (**a**) Cell lysates were analyzed by WB for phospho-Ser^792^Raptor (pRaptor), Raptor, phospho-Ser^555^ULK (pULK), ULK1, and actin. Results are representative of 3 independent experiments. (**b**) Visualization of mKate2-LC3 in neurons treated with GSK621 (GSK, 10▒µM, 48▒h), BafilomycinA1 (BafA1, 100▒nM, 24▒h) or both. (**c**) Quantification of mKate2-LC-3 positive vesicles number by cell. Results represent mean▒±▒SEM, n▒=▒30 neurons counted from 3 independent experiments. Scale bar▒=▒25▒µm. (**d-g**) Primary neurons at 20 DIV were pre-treated with the autophagy inhibitor MRT68921 (MRT, 2,5▒µM) for 20▒min prior to GSK621 treatment (GSK, 10▒µM, 48▒h). WB analysis (**d**) and quantification of p62 (**e**), PSD-95 (**f**) and GluN1 (**g**). Results represent mean▒±▒SD, n▒=▒4. (**h**) PSD-95 immunostaining (blue) in neurons expressing mKate2-LC3 (red) and co-treated with GSK (10▒µM, 48▒h) and BafilomycinA1 (BafA1, 100▒nM, 24▒h). (**i**) Fluorescence profile corresponding to the yellow lines in (**h**) and showing the co-distribution of LC3 and PSD-95 in neurons co-treated with GSK621 and Bafilomycin A1. Scale bar▒=▒25▒µm. a.u.▒=▒arbitrary units.

One way ANOVA with Bonferroni’s post hoc test was performed for all experiments. ***p▒<▒0.001 compared to the Ctrl, ^###^p▒<▒0.001 compared to BafA1 condition.

**Additional Fig. 4: Impact of autophagy inhibition on pre-synaptic markers**

(**a-d**) Primary neurons were pre-treated with MRT68921 (MRT, 2.5▒µM) prior to AICAR treatment (1▒mM) for 48▒h at 20 DIV. WB analysis (**a**) and quantification of the ratios SNAP25/actin (**b**), Synapsin Ia/actin (Syn Ia) (**c**), Synapsin IIb/actin (Syn IIb) (**d**). Results represent mean▒±▒SD, n▒=▒3. Neuronal cultures were pre-treated with MRT68921 (MRT, 2.5▒µM) prior to AICAR treatment (1▒mM) for 72▒h at 20 DIV. (**e**) Quantification of pre-synaptic (red), post-synaptic (green) and synaptic (yellow) puncta. Results represent mean▒±▒SD, n▒=▒18 neurons (**f**) Post-synaptic densities extractions were performed to analyze the localization of the different AMPK subunits in synaptic compartments. Analysis by WB of the expression of synaptophysin (SYP), PSD-95, AMPK α1/α2, AMPK α2, AMPK β1/β2 and actin in lysates, non PSD, PSD and cytosolic fractions. One way ANOVA with Bonferroni’s post hoc test was performed for all experiments. **p▒<▒0.01, ***p▒<▒0.001 compared to the Ctrl condition, ^#^p▒<▒0.001 compared to the MRT condition.
